# Deciphering the SUMO code in the kidney

**DOI:** 10.1111/jcmm.14021

**Published:** 2018-12-01

**Authors:** Zhen Yang, Yuming Zhang, Shiren Sun

**Affiliations:** ^1^ Department of Nephrology The First Affiliated Hospital of Air Force Medical University Xi'an Shaanxi China

**Keywords:** kidney diseases, kidney fibrosis, SUMOylation

## Abstract

SUMOylation of proteins is an important regulatory element in modulating protein function and has been implicated in the pathogenesis of numerous human diseases such as cancers, neurodegenerative diseases, brain injuries, diabetes, and familial dilated cardiomyopathy. Growing evidence has pointed to a significant role of SUMO in kidney diseases such as DN, RCC, nephritis, AKI, hypertonic stress and nephrolithiasis. Recently, emerging studies in podocytes demonstrated that SUMO might have a protective role against podocyte apoptosis. However, the SUMO code responsible for beneficial outcome in the kidney remains to be decrypted. Our recent experiments have revealed that the expression of both SUMO and SUMOylated proteins is appreciably elevated in hypoxia‐induced tubular epithelial cells (TECs) as well as in the unilateral ureteric obstruction (UUO) mouse model, suggesting a role of SUMO in TECs injury and renal fibrosis. In this review, we attempt to decipher the SUMO code in the development of kidney diseases by summarizing the defined function of SUMO and looking forward to the potential role of SUMO in kidney diseases, especially in the pathology of renal fibrosis and CKD, with the goal of developing strategies that maximize correct interpretation in clinical therapy and prognosis.

**Key Points**

- SUMO plays a significant role in kidney diseases such as DN, RCC, nephritis, AKI, hypertonic stress, nephrolithiasis, and podocyte apoptosis.
- Our unpublished experiments revealed a crucial role of SUMO in TEC injury and renal fibrosis. Interpreting these results through the lens of recent literature, we have been suggested that SUMO is involved in these processes via regulation of the TGF‐β and HIF‐1α signaling pathways to determine their effects in hypoxia‐induced renal injury.
- SUMO may also affect TEC injury and renal fibrosis by regulating metabolic reprogramming, based on both our experimental data and the latest published studies about EMT, cell cycle arrest, and defective metabolism in the pathogenesis of kidney fibrosis.

## 1 INTRODUCTION

SUMOs are a family of small proteins covalently attached to and detached from other proteins to modify their function within cells. SUMOylation is a post‐translational modification involved in a series of cellular processes, including nuclear‐cytosolic transport, transcriptional regulation, apoptosis, protein stability, stress response, and cell cycle progression.^1^ Meanwhile, an increasing number of clinical cases have connected SUMO modification to many important diseases such as cancers, neurodegenerative diseases, brain injuries, diabetes, and familial dilated cardiomyopathy.^2^, ^3^, ^4^, ^5^, ^6^, ^7^ However, the function of SUMO in the kidney and the relationship between SUMO and kidney diseases are still unclear. So far, only a few related studies have been performed, therefore, we will review these publications and attempt to decipher the SUMO code in the kidney.

## 2 SUMO MODIFICATION

### 2.1 Post‐translational modifications and SUMO

Post‐translational modifications (PTMs) represent a mechanism by which complex biological processes are orchestrated dynamically at the system level. PTMs can alter protein structure and provide functional diversity to cells in terms of physiological function. Moreover, alterations in protein PTMs have been involved in numerous human disease pathogenesis.^8^, ^9^ These covalent modifications, including phosphorylation, acetylation, ubiquitination, and SUMOylation, rely on a series of enzymes for reversible conjugation/deconjugation that respond promptly to the requirements of the cell state and are essential for the dynamic regulation of cellular processes.^10^ The covalent conjugation of ubiquitin (Ub) and Ub‐like molecules (Ubls), the best known of them being SUMO, has been intensely scrutinized in the last few years.

The ubiquitin‐related protein SUMO‐1 was initially discovered in studies on nuclear import in mammalian cells as a covalent modification of RanGAP1.^11^, ^12^, ^13^, ^14^, ^15^, ^16^ Subsequently, the other three SUMO isoforms, termed SUMO‐2, SUMO‐3, and SUMO‐4, have been defined one after another in mammals. SUMO‐2 and SUMO‐3 are very similar in sequence and are therefore sometimes collectively referred to as SUMO‐2/3.^17^ On the other hand, SUMOylation is a dynamic process that is readily reversed by a family of ubiquitin‐like protein‐specific proteases (Ulp) in yeast and Sentrin/SUMO‐specific proteases (SENPs) in humans.^18^ Precursor SUMO is processed by SENPs to generate mature SUMO,^19^ which is subsequently conjugated to target proteins through an enzymatic cascade involving the dimeric E1‐activating enzyme SAE1/2, the E2 conjugation enzyme Ubc9 and several catalytic E3 enzymes.^20^ SUMOylation is often found to target lysines within the canonical consensus motif [VIL] KxE in proteins.^21^, ^22^

Since its discovery in the mid‐1990s, SUMO‐related post‐translational modification has been shown to affect a vast range of proteins in many pathways.^23^, ^24^, ^25^, ^26^, ^27^ SUMOylation can regulate many aspects of normal protein function, including interactions, subcellular localization, activity, stability, and partnering, and it has been shown to modulate an increasing number of cellular pathways.^28^, ^29^, ^30^

### 2.2 The physiological and pathological function of SUMO

SUMO is essential for the viability of all eukaryotic life, except for some species of yeast and fungi.^28^ Ubc9 knockout mice die at the early post‐implantation stage as a result of chromosome condensation and segregation defects.^31^ SUMO‐2 has been found to be indispensable for the embryonic development of mice, whereas SUMO‐1 and SUMO‐3 knockout mice are still viable.^32^ Generally, SUMOylation is a critical event in the dynamic regulation of protein stability, location, structure, function, activity, and interaction with other proteins and as such plays an important role in organism complexity. In addition, emerging research has revealed that SUMO regulates many aspects of cellular physiology to maintain cell homeostasis, both under normal conditions and during cell stress.^33^

Numerous studies have linked SUMO modification to many important diseases such as cancers, neurodegenerative diseases, brain injuries, diabetes, and familial dilated cardiomyopathy.^2^, ^3^, ^4^, ^5^, ^6^, ^7^ Further, genetic and cell biological experiments indicate a critical role of balanced SUMOylation/deSUMOylation in proper cardiac development, metabolism, and stress adaptation.^34^ Recently, abnormal SUMOylation has also emerged as a new feature of heart failure pathology.^35^ In addition, SUMO has been shown to regulate APP and tau and may modulate other proteins implicated in Alzheimer's disease (AD), which may be a novel neuroprotective approach for AD.^36^

## 3 SUMO IN KIDNEY DISEASES

It was first reported that the SUMOylation E2 UBC9 was expressed in the kidney.^37^ Then, SUMO4 was implicated in the pathology of diabetic nephropathy.^31^ Emerging evidence has indicated that SUMOylation and deSUMOylation have roles in more nephropathy diseases such as renal dysgenesis, renal carcinoma, glomerular disease, podocyte apoptosis, renal medulla hypertonicity, acute kidney injury, and nephrolithiasis^38^, ^39^, ^40^, ^41^, ^42^, ^43^, ^44^ (Table 1).

**Table 1:** The involvement of SUMOylation in kidney diseases

Related fields	Study (Author and year)	SUMO effection
Renal dysgenesis	Kloeckener‐Gruissem et al (2005)^38^	A new and reclassified ICF patient without mutations in DNMT3B interacts with proteins SUMO‐1 and UBC9
DN	Guo et al (2004)^31^	TheM55V variant of SUMO4is associated with T1D
	Noso et al (2005)^45^	SUMO4 is associated with T1D in Asian populations with heterogeneity among diverse ethnic groups
	Wang et al (2008)^46^	SUMO4 is a T1D susceptibility gene in multiple Asian populations while controversial observations in Caucasians
	Lin et al (2007)^39^	SUMO4 M55V variant is associated with diabetic nephropathy in T2D
	Gao et al (2014)^49^	Ubiquitination and SUMOylation may contribute to the pathology of DN
RCC	Bertolotto et al (2011)^111^	A SUMOylation‐defective MITF germline mutation predisposes to melanoma and renal carcinoma
	Morell‐Quadreny et al (2011)^115^	The IHC expression of Ubiquitylation and SUMOylation cannot be considered evaluable markers for discriminating the effects of long‐term, low‐dose IR exposure in cRCC carcinogenesis
AKI	Lu et al (2013)^59^	SUMOylation of PPARγ by RGL Prevents LPS induced NCoR Degradation Mediating Down Regulation of Chemokines Expression in RPTCs
	Chen et al (2014)^60^	Inflammatory factor‐specific SUMOylation regulates NF‐κB signalling in glomerular cells from diabetic rats
	Guo et al (2015)^43^	SUMOylation occurs in AKI and plays a cytoprotective role
Hypertonic renal medulla and Hephrolithiasis	Kim et al (2014)^42^	SUMOylation modulates the activity of TonEBP in the hypertonic renal medulla to prevent excessive action of TonEBP
	Yusof et al (2015)^44^	An increase in serum concentrations of DNase I/II and E3 SUMO‐protein ligase NSE2 level can be used as indicators for the diagnosis of kidney injury in patients with nephrolithiasis
CKD	Wang et al (2014)^41^	Inhibition of p53 deSUMOylation Exacerbates Puromycin Aminonucleoside Induced Apoptosis in Podocytes
	Tossidou et al (2014)^82^	SUMOylation participates in the tight orchestration of nephrin turnover at the slit diaphragm
	Wang et al (2015)^83^	Podocytes protect glomerular endothelial cells from hypoxic injury via deSUMOylation of HIF‐1α signalling

### 3.1 SUMO and diabetic nephropathy (DN)

Although many connections have been found between SUMO and human diseases, limited direct evidence has been shown linking SUMO and kidney diseases. Over a decade ago, it was first discovered that the SUMOylation E2 UBC9 was highly expressed in the kidney.^37^ Subsequently, SUMO4 was implicated in the pathology of DN.^31^ Further studies, however, were inconsistent in associating SUMO4 with type 1 diabetes (T1D). Despite controversial observations in Caucasians, the M55V polymorphism was significantly associated with T1D in Asian populations, which implied heterogeneity in the genetic effect of the SUMO4/MAP3K7IP2 locus on T1D among diverse ethnic groups.^45^, ^46^ Next, two primary breakthroughs at the molecular level have since occurred: first, it was found that glucose may activate NF‐κB inflammatory signalling through IκBα SUMOylation in rat mesangial cells^47^; then, it was also revealed that high glucose may activate TGF‐β/Smad signalling through SUMOylation of Samd4 by SUMO2/3 in mesangial cells.^48^ Overall, crosstalk between ubiquitin and SUMO was implicated in the progression of DN through their regulation of several signalling pathways, including NF‐κB, TGF‐β, Nrf2‐oxidative stress, and MAPK.^49^ These findings may reveal a new point of therapeutic intervention for DN and inspire new treatment strategies for the disease.

### 3.2 SUMO in the hypertonic renal medulla and nephrolithiasis

In addition to the cases described above, there have recently been some attracting findings also relating SUMO to kidney diseases. For example, TonEBP is a DNA‐binding transcriptional enhancer that enables cellular adaptation to hypertonic stress by promoting expression of specific genes.^50^ TonEBP expression is very high in the renal medulla because local hypertonicity stimulates its expression.^51^ Further study has shown that SUMOylation inhibits TonEBP in a manner that is dose‐dependent but independent of the site of SUMO conjugation. In this work, SUMOylation inhibited transactivation without affecting nuclear translocation or DNA binding. These data suggest that SUMOylation modulates TonEBP activity in the hypertonic renal medulla to prevent excessive TonEBP activity.^42^

Besides, several studies have found abnormal DNase levels in many diseases. High serum DNase concentrations were found in patients with renal failure,^52^ acute lymphoblastic leukemia,^53^ and genitourinary cancer.^54^ Malaysian scientists recently found that mean levels of sera NSMCE2 were significantly higher (*P* < 0.01) in patients compared to the control group. The activities of serum DNase I and II were also significantly elevated in nephrolithiasis patients (*P* < 0.01) compared to controls. Eventually, they discovered and supposed that both increased serum concentrations of DNase I/II and E3 SUMO‐protein ligase NSE2 levels could be used as indicators for diagnosing kidney injury in patients with nephrolithiasis.^44^

### 3.3 SUMO in acute kidney injury (AKI)

Investigations performed in human renal proximal tubular cells (PTCs) showed that Rosiglitazone (RGL), a synthetic agonist for peroxisome proliferator activated receptor γ (PPARγ), which exhibits potent anti‐inflammatory activity by attenuating local infiltration of neutrophils and monocytes in the renal interstitium,^55^, ^56^, ^57^, ^58^ activated the SUMOylation of PPARγ and thus suppressed NCoR degradation and the activation of NF‐κB signalling, which in turn decreased chemokine expression. These results unveiled a new molecular mechanism triggered by RGL for prevention of tubular inflammatory injury and fibrosis.^59^ In a more recently published research, SUMO4 was also suggested to play a role in regulating NF‐κB signalling in glomerular cells. Cytokines like (TNF)‐α, NF‐κB (p65), and IκBα have been suggested to have a unique effect in regulating the SUMOylation of NF‐κB.^60^ Additionally, a report about the contribution of SUMOylation to the pathogenesis of acute kidney injury (AKI), formerly termed acute renal failure (ARF), which is a major kidney disease associated with high mortality (N50%),^61^, ^62^ has recently come to our attention. In this report, cisplatin‐induced SUMOylation in rat kidney proximal tubular cells (RPTCs), was diminished by two antioxidants (*N*‐acetylcysteine and dimethylurea), supporting a role of oxidative stress in the activation of SUMOylation. Further, SUMOylation by SUMO‐2/3, but not SUMO‐1, was partially suppressed by pifithrin‐alpha (a pharmacological inhibitor of p53), supporting a role of p53 in SUMOylation by SUMO‐2/3.^63^ Taken together, these results supplied the first evidence of SUMOylation in AKI and suggested that SUMOylation might play a cytoprotective role in kidney tubular cells.

### 3.4 SUMO in renal fibrosis and CKD

The high prevalence and burden of CKD have been well‐established, and it has emerged as a major threat to public health as a result of its 10.8% incidence rate. It is generally accepted that all primary causes of CKD share a common pathogenetic pathway of progressive injury as a result of the destructive consequences of scarring (fibrosis). Renal fibrosis has been shown to be the most reliable predictor of progression to end‐stage renal failure. Thus, understanding the fundamental pathways that lead to renal fibrosis is essential to developing better therapeutic options for human CKD. Notably, oxygen tension is maintained by the balance between oxygen supply and consumption, while chronic oxygen deprivation in CKD takes place in multiple processes when this balance is broken, including decreased oxygen supply because of glomerular damage, imbalanced vasoactive substances, peritubular capillary rarefaction, and increased oxygen consumption.^64^, ^65^, ^66^, ^67^

**Figure 1:**
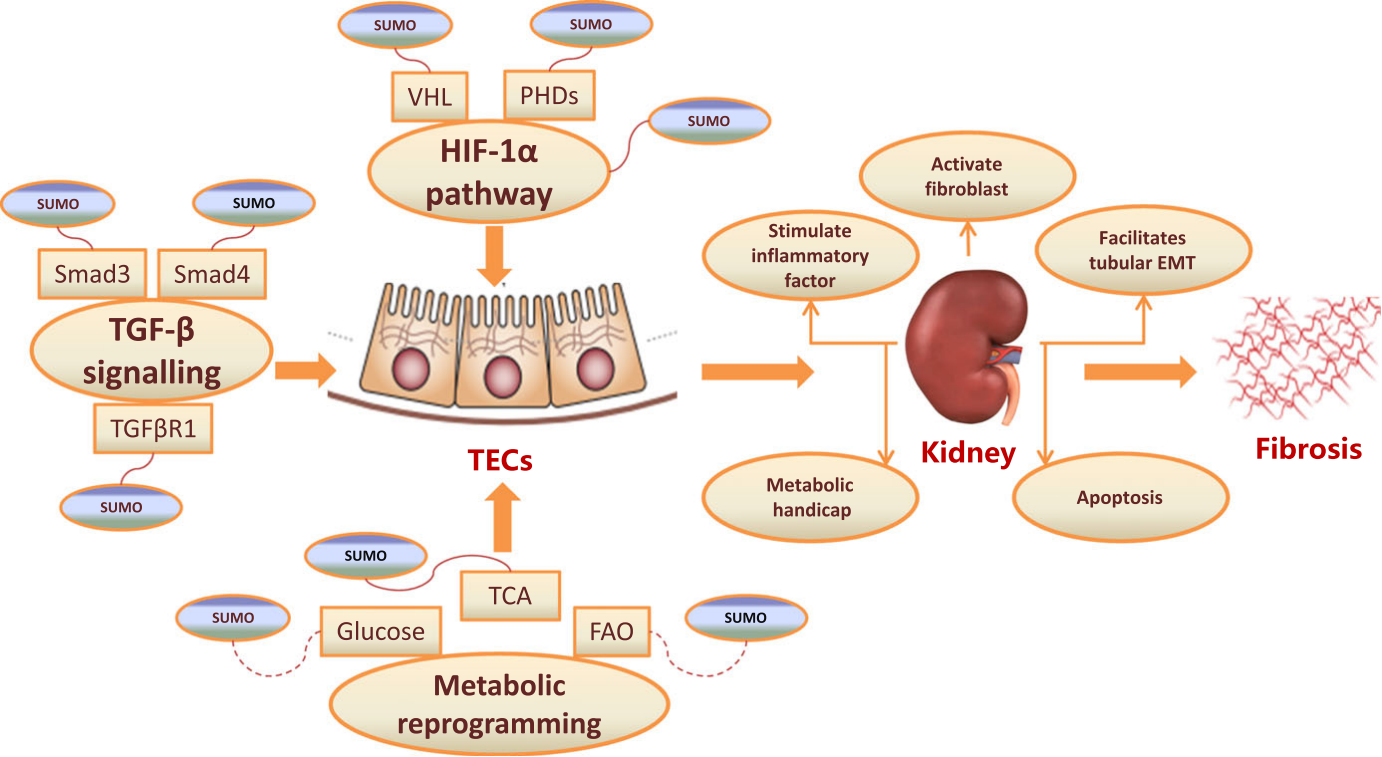
The role of SUMO in kidney fibrosis. SUMO may be involved in the progression of kidney diseases via at least three pathways: the SUMOylation of the set of TGF‐β signalling (Smad3, Smad4 and TGFβR1), the SUMOylation of the set of HIF‐1α pathway (HIF‐1α, VHL and PHD), and the competitive processes of the Metabolism (Glucose, FAO and TCA) and subsequently stimulates inflammatory factor, activates fibroblast, facilitates tubular EMT, forms metabolic handicap, and induces apoptosis, which finally leads to the kidney fibrosis

On the other hand, SUMOylation has also been shown to be one of the main events responsible for hypoxia. The first evidence associating protein SUMOylation with altered cellular metabolic states came in 2003 with the demonstration of increased global protein SUMOylation in vitro under conditions of decreased oxygen tension (hypoxia).^68^ This result was supported by further studies demonstrating increased patterns of protein SUMOylation in mouse brain and heart following exposure to whole animal hypoxia.^69^ Initial studies investigating the role of hypoxia in regulating protein SUMOylation revealed HIF itself to be a target for SUMOylation.^70^ While, whether SUMOylation increases or decreases HIF‐dependent transcription remains controversial.^71^, ^72^, ^73^, ^74^ Anyhow, modulating HIF and its transcriptional activity is likely to be the primary mechanism by which SUMOylation impacts tissue survival during hypoxia, connecting SUMO with renal fibrosis and CKD (Figure 1).

Recently, SUMO has also been implicated in podocyte apoptosis. Clinical and experimental studies have revealed that decreased podocyte number is closely associated with the initiation of glomerulosclerosis and contributes to CKD progression.^75^, ^76^, ^77^, ^78^ Apoptosis is a major cause of reduced podocyte numbers in glomerular diseases,^79^, ^80^, ^81^ and the SUMO protease SENP1 was implicated to be essential for preventing podocyte apoptosis, at least partly through regulating the function of p53 protein via deSUMOylation.^41^ Moreover, inhibition of SUMOylation has been shown to cause reduced membrane expression of nephrin, suggesting that reversible SUMOylation participates in the tight orchestration of nephrin turnover at the slit diaphragm.^82^ Further research has indicated that hypoxia might promote HIF‐1α stabilization and activation by increasing SENP1 expression in podocytes, which induces GEnCs survival and angiogenesis to resist hypoxia.^83^ Thus, deSUMOylation of HIF‐1α signalling is likely to be a novel therapeutic target for treating hypoxic renal disorders.

Meanwhile, TGF‐β/Smads pathway, which signals through Smad‐ and non‐Smad‐dependent pathways and leads to multiple biological effects, is considered the most ubiquitous profibrotic cytokine in progressive renal fibrosis.^84^, ^85^, ^86^ Among the Smads family, Smad4, one of the first batches of SUMOylation substrates discovered in the very early stages following the discovery of SUMO,^87^, ^88^, ^89^, ^90^, ^91^, ^92^ is the central Smads mediator of TGF‐β signalling^93^ and eventually leads to significant enhancement of TGF‐β signalling. Besides Smad4, Smad3 also plays a crucial role in the TGF‐β‐mediated signalling pathway, which produces a variety of cellular responses including cell proliferation and differentiation,^94^ and it was demonstrated that PIASy (a E3 of SUMOylation) suppressed TGF‐β signalling by interacting with and SUMOylating Smad3.^95^ In another outstanding work, it was revealed that SUMO was conjugated in a regulated manner to TβRI receptors, which modulate TGF‐β receptor function and define cellular responses to TGF‐β.^96^, ^97^ Therefore, SUMO‐mediated regulation of the TGF‐β/Smads signalling pathway is likely to be another significant mechanism connecting SUMO with renal fibrosis and CKD (Figure 1).

Taking into account these results, plus the importance of hypoxia in the progression of renal fibrosis and CKD, one may conclude that SUMO likely contributes considerably to this progression. Since TGF‐β signalling plays a crucial role in fibrogenesis^98^, ^99^, ^100^, ^101^ and that HIF‐1α is the key mediator in chronic hypoxia‐induced renal injury,^102^, ^103^, ^104^, ^105^ and SUMO is now regarded as the putative regulator of both, it is reasonable to predict that SUMO could regulate the progression of renal fibrosis and CKD via these two pathways.

### 3.5 SUMO and renal cell carcinoma (RCC)

Another sensational discovery of SUMO‐kidney was recently made in melanoma and renal cell carcinoma (RCC).^106^ The microphthalmia‐associated transcription factor (MITF) has been proposed to act as a melanoma oncogene^107^; it also stimulates transcription of hypoxia inducible factor (HIF1),^108^ a pathway targeted by kidney cancer susceptibility genes.^109^ It was shown that the germline missense substitution in MITF (Mi‐E318K) had a greater than five‐fold increased risk of developing melanoma, RCC or both cancers. By coincidence, codon 318 was located in a SUMO consensus site (YKXE), and Mi‐E318K severely impaired MITF SUMOylation, which provided insights into the link between SUMOylation and RCC. In the same year, Spanish scientists investigated whether ubiquitylation and SUMOylation were involved in conventional renal cell carcinogenesis associated with chronic, long‐term, persistent low doses of ionizing radiation (IR) in patients living for more than 20 years in cesium‐137 (^137^Cs)‐contaminated areas after the Chernobyl accident in Ukraine.^110^ However, they did not consider the immunohistochemical expression of ubiquitylation and SUMOylation as valuable markers for discriminating the effects of long‐term, low‐dose IR exposure in RCC carcinogenesis.

In total, these studies have only scratched the surface of this area of research, and the mechanism governing outcome mediated by SUMOs still needs to be elucidated. Meanwhile, many questions and doubts remain to be addressed somehow, and we are about to discuss them in the next section.

## 4 DISCUSSION AND PERSPECTIVE

The first examples of kidney disease‐associated mutations in SUMOylation sites and/or dysregulation are beginning to emerge. SUMO seems to contribute to physiological complex assembly and can, in some cases, prevent pathological protein aggregation. One possible function of ATP‐dependent reversible SUMOylation is to behave like a chaperone. However, it is still difficult to decipher the SUMO code in kidney disease based on the limited evidence currently available, and tools for identifying and analysing endogenous SUMO targets in complex nephridial tissues in the context of physiological processes need further improvement as well.

It is possible that the SUMO proteome would exhibit global changes in response to cellular stress (eg, hypoxia), but the purpose of these changes is not clear. The exact networks and pathways activated and inhibited in a coordinated fashion via this “SUMO switch” are still mysterious. Regarding renal fibrosis and CKD, it is reasonable to anticipate that SUMO participates in this process via the TGF‐β and HIF‐1α signalling pathways. Notably, an emerging concept in SUMOylation is the requirement for simultaneous (de)modification of multiple targets (group SUMOylation^111^) that are involved in the same biological process. Though inhibiting any single modification may not have any obvious consequences, inhibiting modification of several pathway components does. It seems that both TGF‐β signalling and the HIF‐1α pathway likely participate in this phenomenon, with SUMOylation occurring on Smad3, Smad4, and TGFβR1 in TGF‐β signalling and on HIF‐1α, VHL, and PHD in the HIF‐1α pathway. Nevertheless, more work will be needed to unify our understanding of the effect of SUMOylation on both signalling pathways.

In addition to the pathways above, we must also emphasize the potential role of SUMOylation in metabolic reprogramming in hypoxia‐induced renal injury, as a result of recent discoveries linking the pathogenesis of kidney fibrosis to EMT, cell cycle arrest, and defective metabolism.^112^, ^113^, ^114^, ^115^, ^116^ Substantial evidence is building that SUMOylation of key regulators of metabolism may represent a newly discovered strategy by which cells protect themselves during metabolic stress. There are at least three points at which SUMO may regulate metabolism under conditions of metabolic stress: First, SUMOylation may regulate glucose transporters and thus glucose entry into the cell. Second, mitochondrial morphology may also be under the control of SUMOylation. Third, the transcriptional regulator HIF, which regulates the expression of a range of metabolic genes, may also be a functional target for SUMOylation.^33^ Furthermore, data from our laboratory suggest that SUMO may also take part in the TCA cycle (data unpublished). Taken together, metabolic insights combined with new findings in renal fibrosis recently support a model whereby SUMO exerts influence on renal fibrosis through regulation of metabolic reprogramming (Figure 1).

## 5 CONCLUSIONS

In conclusion, SUMOylation contributes to numerous pathways in developing and adult organisms, and an increasing number of diseases are being associated with a failure to appropriately regulate SUMOylation. Though some cases have implicated SUMOylation in kidney function, uncovering the mechanisms accounting for this role remains a formidable challenge in the field. Fortunately, an increasing number of studies of SUMO function in the kidney have recently been carried out, which will probably unveil the role of the SUMO pathway in the progression of renal pathology soon. In this review, we attempt to delineate the contributions of SUMOylation in the development of kidney diseases by summarizing the defined function and behaviour of SUMO and predicting its potential role in kidney diseases, particularly in the pathology of renal fibrosis and CKD, with the goal of developing strategies that maximize correct interpretation in clinical therapy and prognosis.

## GLOSSARY

**SUMO**. Small Ubiquitin‐like Modifier (or SUMO) proteins are a family of small proteins that are covalently attached to and detached from other proteins in cells to modify their function.

**Renal fibrosis**. Renal fibrosis of the glomerular and tubule interstitial compartments is a common feature of chronic kidney disease leading to end‐stage renal failure, which involves a number of pathologic mechanisms, including cell death and inflammation.

**CKD**. Chronic kidney disease (CKD) is progressive loss in kidney function over a period of months or years.

## CONFLICT OF INTEREST

The authors have no conflict of interest to disclose.
